# Dates, Polyunsaturated Fatty Acids, Dietary Fiber, and Probiotics as Potential Complementary Modulators of Gut Microbiota and Oxidative Stress: An Integrative Review

**DOI:** 10.3390/antiox15091097

**Published:** 2026-08-31

**Authors:** Hassan Barakat, Ahmed H. Bahloul, Ahmed A. H. Abddelatif

**Affiliations:** 1 Department of Food Science and Human Nutrition, College of Agriculture and Food, Qassim University, Buraydah 51452, Saudi Arabia; 2 Food Technology Department, Faculty of Agriculture, Benha University, Moshtohor 13736, Egypt; ahmed.bahloul@fagr.bu.edu.eg; 3 Department of Pharmaceutics, College of Pharmacy, Qassim University, Buraidah 52571, Saudi Arabia; a.abdellatif@qu.edu.sa

**Keywords:** dates, ω-3 and ω-6 fatty acids, dietary fiber, probiotics, gut health, balanced nutrition, functional foods, polyphenols

## Abstract

This integrative review examines the potential of dates, omega-3 (ω-3) and omega-6 (ω-6) polyunsaturated fatty acids (PUFAs), dietary fibers (DFs), and probiotic bacteria to support gut health through complementary nutritional pathways. The literature was searched in PubMed, Web of Science, Scopus, and Google Scholar for English-language studies published through June 2026. Dates contain fermentable substrates, including DFs and non-digestible oligosaccharides, that can serve as growth substrates for beneficial gut microbes. However, the whole fruit has not been formally established as a prebiotic under current consensus definitions. ω-3 and ω-6 fatty acids contribute to membrane integrity, eicosanoid synthesis, immune regulation, and inflammatory signaling, with effects influenced by dietary balance. DFs support gastrointestinal function, glycemic regulation, lipid metabolism, and colonic fermentation, including short-chain fatty acid (SCFA) production, whereas probiotics may exert strain-specific effects on microbial balance, barrier function, immune responses, and host metabolism. Together, these components may influence gut microbiota, oxidative stress, inflammation, and metabolic homeostasis. However, the evidence is heterogeneous, combined interventions are scarce, and several proposed effects rely mainly on preclinical studies or surrogate human outcomes. Thus, potential collaborative effects and disease-prevention implications remain unconfirmed. Well-designed human trials using standardized preparations, realistic intake levels, and clinically relevant outcomes are needed to clarify their combined effects.

## 1. Introduction

Adequate nutritional balance is essential for reducing the risk of chronic disease and maintaining metabolic and digestive health across all stages of life [1]. Current nutrition research increasingly emphasizes food structure, bioactive compounds, and interactions among dietary components, the gut microbiota, and the host, rather than macronutrient adequacy alone. This perspective has driven growing interest in functional foods and dietary patterns that extend beyond basic nutritional requirements [2]. Within this framework, dates, ω-3 and ω-6 PUFAs, DFs, and probiotics are relevant because they act on overlapping gut-related pathways, including modulation of microbiota composition, intestinal barrier function, immune responses, and cardiometabolic risk [2,3].

Dates (*Phoenix dactylifera* L.) are among the most culturally significant fruits worldwide, cherished for their nutritional value and historical medicinal uses [4]. They contain natural sugars, DFs, minerals, vitamins, and a wide variety of phenolic compounds, which contribute to antioxidant and anti-inflammatory activity [5]. Dates are energy-dense fruits, but their physiological effects vary by variety, ripeness, portion size, and dietary context [6]. Consequently, there has been increasing interest in their potential contribution as complete foods to diet quality, satiety, glycemic modulation, and gut health [5]. Essential ω-3 and ω-6 PUFAs are important for membrane structure, the production of eicosanoids, and immune regulation [7]. However, modern diets are often characterized by an imbalanced intake, particularly an excess of ω-6 relative to ω-3, which may affect inflammatory signaling and cardiometabolic outcomes [8]. DFs are also an important part of a healthy diet and one of the most stable dietary factors influencing gut function [9]. They contribute to glycemic control, satiety, and lipid metabolism. At the same time, fermentable fractions serve as substrates for the gut microbiota, producing SCFAs, which are important for barrier integrity, immune function, and host metabolism [10,11]. These properties position fiber as an important mediator of diet–microbiota interactions rather than merely a structural dietary constituent [12]. Probiotics are also a hot topic in science, as they can help improve the balance of gut microbiota and thus support host health when taken in adequate amounts [13,14]. The most-studied probiotic genera include Lactobacillus, Bifidobacterium, and certain yeasts, such as *Saccharomyces boulardii* [15]. Their benefits are strain-specific and may include reinforcement of barrier function, suppression of pathogenic organisms, modulation of immune activity, and improvements in certain digestive and metabolic outcomes [16]. However, their relevance is enhanced when fermentable dietary substrates, particularly fiber-rich foods, are present, which may support microbial persistence and activity [17].

**Figure 1:**
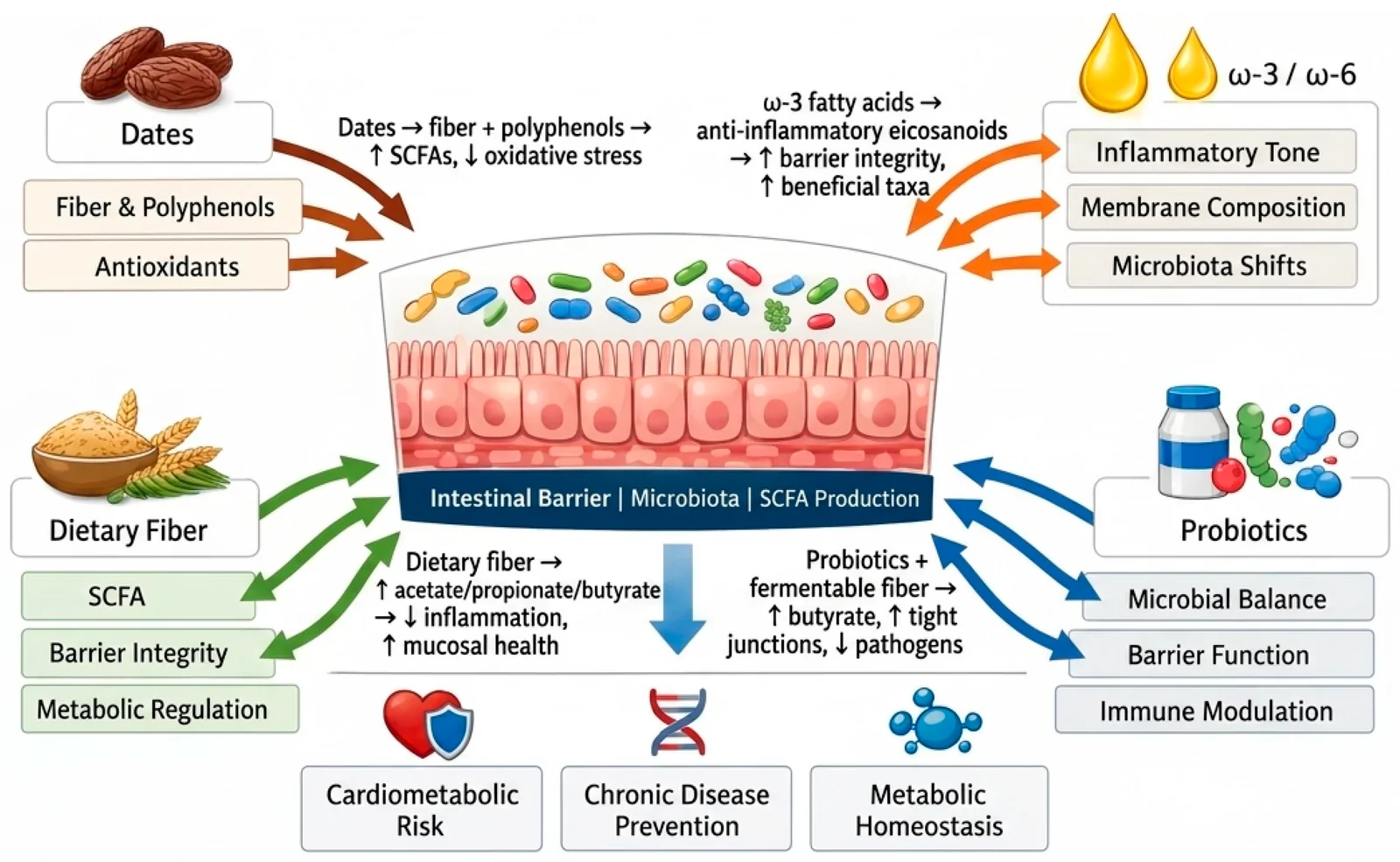
Mechanistic framework illustrating how dates, ω-3/ω-6, DFs, and probiotics converge on gut micro-biota, SCFAs production, barrier integrity, oxidative stress, and inflammation; solid and dashed lines represent human and preclinical evidence, respectively.

Rather than acting as individual nutrients, they function as a nutrient network. Interactions between these elements are essential for balanced nutrition and gut health [18]. As shown in Figure 1, each component is linked to specific mechanisms, such as dates to fiber and polyphenols, ω-3 to anti-inflammatory eicosanoids, fiber to SCFA production, and probiotics to barrier strengthening and competitive exclusion of pathogens. The combined effects of these components are not due to isolated nutrient actions, but rather to their convergence on microbiota–SCFAs–barrier–inflammation pathways. Additionally, Figure 1 was used as the organizing framework for the evidence synthesis in this review. Specifically, the effects of dates, ω-3/ω-6 fatty acids, DFs, and probiotics were examined across five interconnected biological domains: gut microbiota composition and activity, microbial production of SCFAs, intestinal barrier integrity, oxidative stress, and inflammation. The framework also distinguishes between evidence derived from human studies and evidence based primarily on animal or in vitro models. This distinction is important because mechanistic plausibility does not necessarily demonstrate a clinically meaningful effect in humans. Accordingly, the following sections discuss each dietary component in relation to these five pathways and identify areas where human evidence remains inconsistent, indirect, or insufficient.

Oxidative stress is considered a distinct domain because dysbiosis, barrier dysfunction, inflammation, lipid peroxidation, and impaired antioxidant defenses may interact. Evidence varies across chemical, experimental, systemic, and intestinal measures. Dates, ω-3/ω-6 fatty acids, DFs, and probiotics may act through complementary pathways that affect the microbiota, SCFA production, barrier integrity, inflammation, and oxidative stress, yet their combined effects remain insufficiently synthesized. Therefore, this review focuses on dates, ω-3/ω-6 fatty acids, DFs, and probiotics as adjunct factors influencing gut–metabolic health through microbiota composition, SCFA production, barrier integrity, and inflammation. Dates contain fermentable fiber and polyphenols that support microbial activity and antioxidant capacity, while ω-3/ω-6 fatty acids regulate membrane function and inflammatory signaling. DFs drive colonic fermentation and metabolic regulation. Probiotics modulate microbial balance, barrier function, and immune responses, often with fiber. Accordingly, this integrative review synthesizes current evidence on the composition, mechanisms of action, and health effects of dates, ω-3/ω-6 fatty acids, DFs, and probiotics as interactive nutritional modulators. It highlights (i) their nutritional and bioactive functions in modulating gut microbiota and gut health, (ii) common pathways of microbial dynamics, oxidative stress, inflammation, and metabolic regulation, and (iii) translational perspectives and research priorities in advancing precision nutrition.

## 2. Search Strategy and Methodology

This extended review includes the latest evidence on the role of dates, ω-3 and ω-6, DFs, and probiotic bacteria within the framework of balanced nutrition and gut health. The literature was searched systematically in PubMed, Web of Science, Scopus, and Google Scholar, and supplemented by targeted screening of leading journals in nutrition, food science, and microbiome. The search was limited to English-language articles published until June 2026. It included a combination of controlled vocabulary and free-text terms for dates (e.g., *P. dactylifera* L., date fruit), ω-3 and ω-6 (e.g., PUFA, ω-3, ω-6, fatty acid ratio), DFs (e.g., prebiotics, fermentable fiber, SCFAs), probiotics (e.g., Lactobacillus, Bifidobacterium, synbiotics), and gut-related outcomes (e.g., gut microbiota, intestinal barrier, inflammation, oxidative stress, cardiometabolic risk). Additional relevant publications were identified by hand-searching reference lists of key articles and recent reviews.

Peer-reviewed original research, clinical trials, mechanistic studies, and narrative or systematic reviews on the four focal components in humans, animal models, or in vitro systems were eligible, with preference given to studies with clear methods, adequate sample size, and relevant endpoints, and greater weight assigned to work published up to June 2026. Studies using whole foods (e.g., whole dates, fish, fiber-rich foods, fermented foods) and those using isolated supplements or extracts (e.g., date extracts, fiber supplements, fish/algae oil capsules, probiotic supplements) were identified and interpreted separately wherever possible. Differences in formulation (whole food vs. supplement/extract) were considered when synthesizing the evidence and grading its translational relevance. Non-peer-reviewed material, conference abstracts, editorials, and poorly characterized studies were excluded. Studies were grouped by design and, where possible, by outcome domain. Human clinical or observational evidence was distinguished from preclinical findings, which were interpreted cautiously as mechanistic and hypothesis-generating. Given substantial heterogeneity across designs, populations, interventions, and outcomes, no meta-analysis was performed; instead, a qualitative synthesis was conducted to compare the quality of the evidence, consistency, and translational relevance. Evidence was qualitatively graded as strong, moderate, or emerging. Strong evidence reflects consistent findings from multiple well-designed human studies; moderate evidence reflects supportive but heterogeneous human data, often limited by sample size, duration, or surrogate outcomes; and emerging evidence relies mainly on preclinical or preliminary human findings. These categories indicate translational confidence, not formal risk-of-bias assessment.

## 3. Bioactive Components in Dates

### 3.1. Nutritional and Bioactive Composition of Dates

Dates have a high carbohydrate content, with simple sugars such as glucose, fructose, and sucrose as the major energy sources [19]. They are also a source of DFs, particularly insoluble fiber, which supports gastrointestinal function and may attenuate postprandial glycemic response. In addition, dates contain a limited amount of protein and very little fat, so they are predominantly carbohydrate-rich whole fruits rather than energy-dense sources of lipids or proteins [19]. Dates are also rich in several micronutrients, including potassium, magnesium, calcium, phosphorus, and iron, as well as trace amounts of B-complex vitamins [20]. These nutrients are essential for energy metabolism, electrolyte balance, neuromuscular function, and antioxidant defense [21]. While the absolute concentration of individual nutrients in cultivars varies greatly, dates are generally considered to be a concentrated natural source of energy and micronutrients, especially in whole-fruit form [6]. Some of the most studied constituents are phenolic acids, such as ferulic, caffeic, and *p*-coumaric acids, and their derivatives, due to their antioxidant potential [22]. Flavonoids, tannins, carotenoids, tocopherols, and phytosterols also contribute to the biological activity and potential health effects of dates and their extracts [23]. The composition and profile of these chemicals are strongly influenced by cultivar, maturity, and storage conditions [24]. The sugar composition, phenolics, and antioxidant activity of softer, riper dates might differ from those of semi-dry or dry types, for instance. This heterogeneity is crucial, as it implies that the biological effects of dates cannot be generalized across all types without consideration of the specific product under investigation [25]. Table 1 summarizes the nutritional and bioactive composition of dates, which varies according to cultivar, ripening stage, and pre- and post-harvest conditions. Dates are a rich source of energy, dietary fiber, micronutrients, and phenolic compounds to support balanced nutrition and gut health [12,19,24].

### 3.2. Health-Promoting Effects of Eating Dates

Moderate consumption of dates appears to support multiple aspects of health, particularly when included within a balanced diet [26]. They present a good antioxidant activity, which is related to their phenolic content and their ability to increase endogenous antioxidant defenses [27]. Dates have also been shown to have anti-inflammatory properties, to enhance lipid profiles under specific conditions, and to favorably modulate oxidative stress indicators [26]. Although dates contain natural sugar, the fiber matrix and polyphenols may decrease glucose absorption and reduce the glycemic response compared with refined sugars [28]. Human studies generally suggest that modest consumption does not adversely affect glycemic control and may be included in healthy diets, including for some people with diabetes when ingested in suitable quantities [29]. However, this observation should not be generalized as a therapeutic recommendation for patients with diabetes or obesity. Although some studies suggest that certain individuals with diabetes may tolerate moderate date consumption, dates remain a carbohydrate- and energy-dense food, and their intake should be considered within the individual’s total carbohydrate distribution, energy requirements, glycemic response, and treatment plan. Patients with diabetes should therefore receive individualized advice from a healthcare professional rather than general recommendations based solely on the potential nutritional benefits of dates. Moreover, replacing low-nutrient snacks with dates may improve satiety and overall diet quality [30].

### 3.3. Mechanisms of Biological Action

Dates contain fermentable substrates with prebiotic supporting properties, although the fruit, as consumed, has not been conclusively shown to meet the formal definition of a prebiotic. Their phenolics and fiber could work via antioxidant, glycemic, and microbiota-mediated mechanisms [31]. Their phenolic compounds can scavenge reactive oxygen species (ROS), minimize oxidative damage, and modify inflammatory signaling associated with obesity, insulin resistance, and cardiometabolic diseases [27]. The fiber content helps reduce carbohydrate absorption, enhance postprandial glycemic control, and promote bowel function [32]. In addition, dates may contain fermentable carbohydrates, including DFs and non-digestible oligosaccharides, which can reach the colon and be metabolized by the gut microbiota. It generates SCFAs via fermentation, which may be associated with intestinal barrier integrity, immunomodulation, and metabolic homeostasis [12].

### 3.4. Oxidative Stress and Redox Modulation

Dates may influence intestinal redox balance through the combined actions of phenolic compounds, micronutrients, and fermentable carbohydrates. Phenolic acids, flavonoids, tannins, carotenoids, and tocopherols can contribute to antioxidant activity by scavenging ROS, chelating transition metals, and limiting lipid and protein oxidation. These compounds may also influence endogenous antioxidant systems, including glutathione-dependent defenses and antioxidant enzymes such as superoxide dismutase, catalase, and glutathione peroxidase. However, the antioxidant capacity measured in chemical assays should not be equated directly with an antioxidant effect in humans because absorption, metabolism, food structure, and microbial transformation determine biological availability [22,23,27].

The fiber and polyphenol fractions of dates may provide complementary redox effects. Fermentable substrates can support microbial production of SCFAs, which may improve epithelial energy metabolism and reduce inflammatory signaling associated with barrier disruption [10,11]. Microbial transformation of date polyphenols may generate metabolites with different antioxidant or signaling properties from those of the parent compounds [31,33]. Through these pathways, dates may indirectly reduce oxidative stress by supporting barrier integrity, limiting exposure to luminal pro-oxidant compounds, and modulating inflammation. Evidence for the antioxidant effects of dates is strongest in chemical, cellular, and animal models, whereas human evidence remains more limited and is generally based on surrogate biomarkers. Reported outcomes include changes in total antioxidant capacity, lipid peroxidation products, protein oxidation, glutathione status, or antioxidant enzyme activity. Differences in cultivar, ripening stage, processing, dose, and whether whole fruit, seed, or concentrated extracts are used substantially limit comparison across studies [24,25,34]. Accordingly, dates should be described as a promising source of redox-active compounds rather than as a clinically established antioxidant treatment.

### 3.5. Human and Experimental Data

Human and preclinical research have provided evidence of the potential health effects of dates [29]. Clinical investigations demonstrate that moderate intake is generally well tolerated and does not adversely affect glycemic management, with some reports of benefits in lipid profiles, antioxidant status, and inflammatory markers. However, results are inconsistent [34]. In comparison, preclinical data support plausibility; direct translation to normal food consumption remains limited. Animal and in vitro studies have shown that chemicals derived from dates have antioxidant, anti-inflammatory, and metabolic properties, including decreases in oxidative stress and regulation of signaling pathways [35]. However, many of these investigations are conducted using concentrated extracts or under controlled experimental conditions that may not fully represent typical food consumption [35].

Table 2 summarizes data from human, animal, and in vitro research, including major findings and methodological issues. Overall, the data is positive but hampered by small trials, short durations, and varied effects. Many trials are limited by small sample sizes, brief intervention periods, objectives that focus on surrogate indicators rather than clinical outcomes, and uneven reporting of microbiota-related metrics. Furthermore, heterogeneity in research designs, limited demographic diversity, and an inability to effectively control for dietary background limit the generalizability of the findings and underscore the importance of larger, well-controlled human trials that employ standardized data preparation.

### 3.6. Significance of Gut Health and a Balanced Diet

Dates are considered useful foods within the framework of balanced nutrition, and not as homogenous therapeutic ingredients. Indeed, the quantity of dates is a major contributor, particularly in groups at risk of obesity, insulin resistance, or dysglycemia [36], and is related to high energy density. Dates can be incorporated into balanced diets in moderate amounts as a whole-food source of fiber and bioactives [37]. They are of particular interest for gut health due to their fermentable substrates and antioxidant phytochemicals that can improve microbial diversity, promote intestinal function, and reduce oxidative stress [33].

Dietary implications should be interpreted in light of the individual’s health status and clinical characteristics. For generally healthy adults, DFs, polyunsaturated fatty acids, and probiotic-containing foods may be incorporated into a varied and balanced dietary pattern in appropriate portions [38,39]. However, these general dietary considerations should not be interpreted as therapeutic recommendations for individuals with diabetes, inflammatory bowel disease, obesity, or other medical conditions. In such populations, the suitability, amount, timing, and form of these foods or supplements may depend on glycemic control, total energy intake, gastrointestinal tolerance, disease activity, medication use, and other clinical factors [40,41]. Therefore, any use of these dietary components as an adjunct to disease management should be individualized and undertaken under the supervision of a physician or qualified dietitian. For individuals with obesity, dates may be used in limited portions as a replacement for confectionery or other nutrient-poor snacks; however, their inclusion alone does not promote weight loss [42]. Because dates are relatively energy-dense, their contribution to total energy intake should be considered within the context of the individual’s dietary pattern and weight-management plan [43]. Evidence concerning dates, probiotics, DFs, or polyunsaturated fatty acids should therefore not be presented as establishing a stand-alone treatment for obesity.

### 3.7. Integrative Implications

As a complete food source, they provide fermentable substrates and bioactive compounds that may enhance microbial activity and preserve antioxidant balance, especially when incorporated into dietary patterns that replace low-nutrient, high-sugar meals [44,45]. Their roles align with synergistic strategies targeting microbiota composition, barrier function, and inflammatory control, contributing to holistic approaches to gut and cardiometabolic health. These considerations relate to balanced nutrition in generally healthy populations and do not constitute disease-specific therapeutic recommendations [42,45].

### 3.8. Link to the Mechanistic Framework

Dates may influence gut health through the combined effects of fermentable fiber and polyphenols, with potential consequences for microbiota activity, SCFA production, barrier integrity, oxidative stress, and inflammation [35]. The redox effects are mechanistically plausible because date phenolics may directly neutralize reactive species or regulate endogenous antioxidant defenses [32]. In contrast, the fiber fraction may act indirectly through microbial fermentation and barrier support. Human evidence mainly supports the tolerability of moderate consumption and selected effects on bowel function, glycemia, and oxidative or inflammatory biomarkers [46]. However, consistent changes in microbiota, fecal SCFAs, validated barrier measures, and clinically meaningful redox outcomes remain insufficiently demonstrated. Fermentable DFs serve as a substrate for colonic microbial fermentation and can promote SCFA production [47,48]. However, fecal SCFA concentrations should not be interpreted as a direct measure of SCFA production, as they are also influenced by colonic absorption, microbial and host utilization, dilution, and intestinal transit [49]. Therefore, fecal SCFA findings should be interpreted with caution and in the context of dietary intake, microbiota, and relevant host metabolic or barrier outcomes.

## 4. ω-3 and ω-6 Fatty Acids

### 4.1. Definition and Classification

The ω-3 family comprises α-linolenic acid, eicosapentaenoic acid, and docosahexaenoic acid, whereas the ω-6 family contains linoleic acid and arachidonic acid [50]. The ω-3 family consists of α-linolenic acid, eicosapentaenoic acid, and docosahexaenoic acid, and the ω-6 family includes linoleic acid and arachidonic acid [51]. These fatty acids are essential membrane components and give rise to lipid mediators that participate in inflammation, immunology, vascular function, and metabolism. Both are important, but their physiological effects are different. ω-3 fatty acids are typically associated with anti-inflammatory and cardioprotective properties, but excessive ω-6 consumption relative to ω-3 may induce a more pro-inflammatory state [52,53]. Therefore, the key nutritional concern is dietary balance rather than individual consumption [54].

### 4.2. Dietary Sources

Marine and plant sources provide ω-3 fatty acids [55]. Fatty fish, such as salmon, sardines, mackerel, and tuna, are good sources of eicosapentaenoic acid and docosahexaenoic acid. Flaxseed, chia seeds, and walnuts provide α-linolenic acid, whereas algal oils are sources of DHA and, in some formulations, EPA [56]. Marine sources remain important because conversion of α-linolenic acid to long-chain forms is limited [57]. ω-6 fatty acids are widely available in vegetable oils (e.g., corn, soybean, sunflower, and safflower) as well as nuts and seeds [52]. In many populations, ω-6 intake exceeds that of ω-3, raising interest in the role of dietary fat quality in inflammation, metabolic health, and gut-related outcomes [8,54].

### 4.3. Health Implications of ω-3 Fatty Acids

Among dietary lipids, ω-3 fatty acids have been among the most studied due to their wide-ranging health relevance [58]. They have been linked to reduced triglyceride levels, improved endothelial function, regulation of inflammatory pathways, and possibly protection against cardiovascular disease [59]. Their effects are also of interest for insulin sensitivity, brain development, cognitive function, and immune regulation [60,61]. From a mechanistic perspective, ω-3 can reduce the production of pro-inflammatory mediators and alter membrane lipid composition, thereby affecting receptor function and intracellular signaling [53]. These actions are pertinent to chronic diseases characterized by low-grade inflammation, such as obesity, type 2 diabetes, and cardiovascular diseases. Moreover, ω-3 consumption can influence the gut microbiota and the intestinal barrier, indicating that the role of ω-3 is not limited to traditional lipid metabolism [62].

### 4.4. Health Aspects of ω-6 Fatty Acids

The main dietary ω-6 fatty acid is linoleic acid, which is essential for membrane structure and the production of biologically active derivatives [63]. Their effects are context-dependent and depend on dose, source, and dietary balance [64]. ω-6 fatty acids are not inherently detrimental and remain necessary for normal physiological function when consumed in appropriate amounts [64]. However, concerns arise when intake is disproportionately high relative to ω-3, particularly within dietary patterns characterized by high consumption of processed foods and lower overall nutritional quality [65,66].

### 4.5. The Balance Between ω-3 and ω-6 in the Diet

Thus, the imbalance may encourage the generation of pro-inflammatory lipid mediators and decrease the availability of ω-3-derived molecules implicated in the resolution of inflammation [63]. One important objective in preventive nutrition is to improve the ω-3/ω-6 ratio [65]. Practical solutions include increasing intake of ω-3-rich foods such as fatty fish, nuts, and seeds, and reducing consumption of ω-6-rich refined oils and ultra-processed foods [52]. Crucially, the goal is not to remove ω-6 but to re-establish a more physiologically appropriate intake pattern [64]. This balance promotes cardiometabolic health, controls inflammation, and can help build a more stable gut microbiome [62].

### 4.6. Implications for Gut Health

These fatty acids may alter gut health via membrane, immunological, and microbial mechanisms [67]. They can modify the inflammatory tone and, consequently, affect the intestinal barrier and mucosal immune responses [68]. Moreover, their dietary choices may indirectly affect microbial composition, particularly given the quality of lipids, fiber, and the whole-food matrix [69]. ω-3 fatty acids have garnered particular attention for their ability to enhance anti-inflammatory profiles in bacteria and reduce intestinal inflammation [39,70]. By contrast, ω-6 fatty acids may have more complex effects depending on its relative abundance and broader dietary context [44]. Moreover, the quality of fatty acids, together with fiber and probiotics, may help create a gut environment that supports microbial diversity and more favorable host responses [71]. The research is extensive but diverse, with most studies providing greater evidence for cardiometabolic indicators than for gut-specific outcomes; ω-3 patterns appear more consistently positive, whereas ω-6 effects depend on dosage and dietary context. Preclinical data support biological plausibility, but human evidence remains only moderate for microbiota diversity and barrier integrity. Table 3 summarizes the major ω-3 and ω-6 fatty acids and highlights the interpretive limitations that complicate the translation of mechanistic and clinical findings into dietary guidance.

### 4.7. Oxidative Stress and Redox Modulation

ω-3 and ω-6 fatty acids influence redox balance primarily through membrane composition, lipid oxidation, inflammatory signaling, and the formation of bioactive lipid mediators [53]. Because polyunsaturated fatty acids contain multiple double bonds, they are susceptible to peroxidation. Their biological effects, therefore, depend on the balance between incorporation into cellular membranes, formation of oxidized lipid products, and the activity of endogenous antioxidant systems [72]. The presence of a PUFA in the diet should not be interpreted as inherently antioxidant or pro-oxidant; the outcome depends on the type of fatty acid, dose, tissue context, the oxidation status of the food source, and the availability of antioxidant protection [52,62].

ω-3 fatty acids may reduce oxidative and inflammatory stress by altering membrane lipid composition and serving as precursors for specialized pro-resolving mediators. These mediators may help terminate inflammation and reduce secondary oxidative injury. ω-3 fatty acids may also influence redox-sensitive transcriptional pathways and interactions among epithelial cells, immune cells, and the gut microbiota [53,58,68]. However, direct evidence of improvement in gut-specific oxidative stress markers in humans remains less developed than evidence for systemic triglyceride levels or selected inflammatory outcomes.

ω-6 fatty acids are physiologically necessary and should not be classified uniformly as pro-oxidant or harmful. Linoleic acid is an essential membrane fatty acid, while arachidonic acid is a precursor for numerous lipid mediators. Under conditions of excessive oxidative stress, inflammation, or high intake of oxidized dietary fats, PUFA peroxidation may generate reactive aldehydes and other products that can affect cellular signaling [72]. Conversely, adequate intake of essential fatty acids within a high-quality dietary pattern supports normal membrane function and physiological signaling [63,64]. The redox consequences of ω-6 intake therefore depend on dietary context, fatty acid quality, concurrent ω-3 intake, and antioxidant status rather than on ω-6 concentration alone [52]. Human evidence is moderate for selected systemic oxidative and inflammatory outcomes, particularly regarding ω-3 intake [62]. Still, it remains limited in assessing intestinal oxidative stress, epithelial redox balance, and microbiota-derived redox signals. Future trials should measure oxidized lipid products, antioxidant capacity, glutathione status, and inflammatory mediators together with microbiota and barrier outcomes.

### 4.8. General Nutritional Significance

ω-3 and ω-6 are important dietary constituents, but their effects on health depend largely on quantity, source, and balance [73]. ω-3 fatty acids are highly valued for their anti-inflammatory and cardiometabolic benefits, and ω-6 fatty acids are essential nutrients; their health effects should be interpreted in the context of total dietary intake, food sources, and overall fatty acid balance rather than by ω-6 intake alone [74]. Thus, modern nutrition advice is increasingly focused on whole dietary patterns that promote a balanced intake of fatty acids rather than correcting individual nutrients [66]. In gut-focused nutrition, ω-3 and ω-6 influence membrane function, inflammatory signaling, and mucosal barrier integrity, and a better balance may support beneficial microbes alongside fiber and probiotics. However, gut-specific evidence remains uneven, as many studies emphasize cardiometabolic outcomes, methodological limitations, and limited population diversity.

### 4.9. Link to the Mechanistic Framework

As shown in Figure 1, ω-3/ω-6 fatty acids primarily influence membrane signaling, lipid mediator formation, inflammation, oxidative balance, and intestinal barrier function, with possible secondary effects on microbiota and SCFA metabolism. ω-3-derived mediators may support the resolution of inflammation and reduce downstream oxidative injury, whereas the redox effects of ω-6 fatty acids depend on fatty acid identity, oxidation status, dose, and dietary context [52,62]. Human evidence is strongest for triglyceride and selected systemic inflammatory outcomes, while effects on gut-specific oxidative stress, microbiota, SCFAs, and intestinal permeability remain inconsistent and context-dependent.

## 5. Dietary Fiber

DFs are a key component of healthy diets, supporting gut function, metabolic regulation, and microbiome composition [75]. They reach the colon largely undigested, where they exert structural, mechanical, and fermentative effects that help regulate satiety, glycemia, and the risk of chronic disease [76].

### 5.1. Categorization of DFs

DFs comprise a diverse group of plant-derived, indigestible carbohydrates and related compounds that differ in solubility, viscosity, and fermentability. Soluble fibers form viscous gels that slow nutrient digestion and absorption, whereas insoluble fibers primarily add bulk to stool and facilitate intestinal transit [77]. Some fibers are highly fermentable and act as substrates for colonic bacteria; others are less fermentable, but aid bowel regularity and stool structure [78]. Also, from a functional perspective, fiber can be classified as prebiotic or non-prebiotic [79]. Common dietary sources include fruits, vegetables, legumes, whole grains, nuts, seeds, and some functional ingredients [80]. Dates are relevant in this respect because they provide a meaningful amount of fiber within a naturally nutrient-rich fruit matrix [6].

### 5.2. Relevance to Diet Quality and Sources of Fiber-Rich Foods

As consistently reported, diets rich in fiber are associated with improved diet quality and more favorable cardiometabolic outcomes [81]. Dietary patterns emphasizing fruits, vegetables, whole grains, legumes, nuts, and seeds provide higher levels of fiber, micronutrients, and a broader range of bioactive compounds than highly processed diets [82]. Fiber is best interpreted within the context of overall food-based dietary patterns [45]. Dates represent a culturally accepted and palatable source of DFs, particularly in regions where they are widely consumed [6]. Although energy-dense due to their natural sugar content, they differ from refined sweets by providing fiber and polyphenols [83]. When consumed in moderation, they may serve as a whole-food alternative to low-fiber, highly processed snacks [84]. Table 4 outlines the main fiber classes and mechanisms, while emphasizing that differences in source, physicochemical properties, and study design influence the consistency and clinical interpretation of reported benefits.

### 5.3. Mechanisms of Action

Fiber promotes health by improving bowel function, slowing absorption, and generating SCFAs. It has been shown to increase stool volume, promote regular bowel movements, and may help reduce constipation [85]. Soluble and viscous fibers may slow gastric emptying and carbohydrate absorption, thus blunting postprandial glycemic excursions. Such effects are especially relevant in subjects with impaired glucose tolerance or type 2 diabetes [86]. The colonic gut bacteria metabolize fermentable fiber to SCFAs, mainly acetate, propionate, and butyrate [69,87]. These metabolites are energy sources for colonocytes, strengthen the intestinal barrier, and modulate immune and inflammatory signaling [40]. DFs may also reduce serum cholesterol by binding bile acids and altering lipid metabolism [88]. Through these pathways, fiber serves as a multifunctional dietary component that supports both gut function and systemic metabolic health [85]. In individuals with inflammatory bowel disease, the tolerability of dates and other fiber-containing foods may vary with disease activity, intestinal strictures, prior surgery, symptom profile, and individual response. The potential benefits of fermentable substrates observed in mechanistic or nonclinical studies should not be interpreted as evidence that dates or supplemental fiber are suitable during active disease or acute gastrointestinal symptoms. Dietary modifications in these patients should be individualized and coordinated with the treating gastroenterologist and dietitian.

### 5.4. Evidence of Health Benefits

Higher fiber intake is associated with better bowel, metabolic, and microbiome outcomes [46]. Increased fiber consumption is associated with improved bowel regularity, better weight control, lower risk of type 2 diabetes, improved lipid profiles, and reduced cardiovascular risk [45], with stronger benefits observed when fiber comes from whole foods rather than supplements [87]. Fiber intake is also associated with favorable shifts in the gut microbiota and increased production of microbial metabolites [89]. In contrast, low-fiber diets are associated with decreased microbial diversity and reduced synthesis of SCFAs, while fiber-rich diets promote a more robust intestinal environment [90]. This microbiome dimension has been a prominent focus of study, particularly in relation to inflammation, obesity, and metabolic dysfunction [87]. Increased fiber consumption has been associated with improved cardiometabolic and gastrointestinal outcomes; evidence varies by fiber type, dosage, and food matrix. Human studies still need longer follow-ups and more functional microbiome assessments to establish which fibers matter most.

### 5.5. Fiber as a Modulator of Microbiome

Fiber is a key substrate for microbiota-driven metabolism and host signaling [18,91]. Microbial growth patterns linked with beneficial bacteria ferment fibers and support host physiological function [92]. This activity also creates compounds that regulate hunger management, glucose homeostasis, immunological function, and mucosal integrity, and help to improve bacterial diversity [90]. The interaction between DFs and gut flora is particularly interesting when evaluated in the context of probiotics [93]. The presence of fiber can improve probiotic survival and activity by providing fermentable substrates that stimulate colonization and metabolism [85]. Thus, fiber is not only a bulking agent but an active participant in gut health [93].

### 5.6. Oxidative Stress and Redox Modulation

DFs may influence oxidative stress primarily through indirect microbiota-mediated and metabolic pathways rather than through direct radical-scavenging activity. Colonic microorganisms convert fermentable fibers into SCFAs, particularly acetate, propionate, and butyrate. These metabolites may support colonocyte energy metabolism, epithelial barrier maintenance, and immune regulation, thereby reducing inflammatory conditions that promote ROS generation [40,69]. Butyrate may be particularly relevant to epithelial redox homeostasis because it supports colonocyte metabolism and may influence antioxidant and inflammatory signaling pathways.

Fiber may also lower oxidative burden by improving glycemic regulation, lipid metabolism, bowel transit, and the removal or dilution of potentially harmful luminal compounds. Improved postprandial glucose control may reduce glycation-related oxidative stress, while favorable effects on lipid metabolism may decrease the availability of substrates for lipid peroxidation. These effects are indirect and depend on fiber type, dose, fermentability, food matrix, and the host’s baseline microbiome [76,85].

Not all fibers exert identical redox effects. Highly fermentable fibers may produce greater SCFA-related effects, whereas less fermentable insoluble fibers may contribute more strongly to stool bulk and transit [94,95]. Some plant fibers are consumed alongside polyphenols and micronutrients, making it difficult to distinguish the effect of the fiber itself from that of the complete food matrix [69,96]. Human studies have reported changes in inflammatory or oxidative stress biomarkers following fiber-rich dietary interventions, but direct evidence of consistent improvements in intestinal oxidative stress remains limited. Therefore, fiber should be regarded as a potential upstream regulator of gut redox balance through fermentation, metabolic control, and barrier support, rather than as a direct antioxidant.

### 5.7. Overall Nutritional Significance

Fiber links whole-food consumption to microbial fermentation and the prevention of chronic illness [45]. It provides essential substrates for microbial activity and the synthesis of SCFAs, which promote gut microbial ecology, barrier function, and metabolic control. It also facilitates interactions with probiotics, ω 3 fatty acids, and polyphenol-rich foods such as dates [17]. Fiber-rich dietary patterns provide a structural basis for integrated food-based approaches to gut health. Evidence for gastrointestinal and metabolic outcomes is somewhat robust, although interpretation is limited by variation in fiber type and amount, dietary sources, and research design and duration. Most studies examine individual fibers and not full food matrices. Differences in microbiome methods and reporting further limit comparability between research. Long-term, well-controlled studies in different groups are needed.

### 5.8. Link to the Mechanistic Framework

DFs provide the most direct link to the microbiota–SCFA–barrier pathway in Figure 1 and may also influence redox balance through these pathways. Their fermentability influences microbial activity and the production of acetate, propionate, and butyrate, which may support barrier integrity, immune regulation, and inflammatory balance. By improving glycemic regulation, lipid metabolism, and epithelial function, fiber may indirectly reduce oxidative stress. Human evidence is relatively consistent for bowel and selected metabolic outcomes, but microbiota, SCFA, barrier, and oxidative-stress responses vary by fiber type, dose, food matrix, and baseline microbiome.

## 6. Probiotics

Probiotics are live microorganisms that, when administered in adequate amounts, confer a health benefit on the host [97,98]. They are extensively studied for their roles in digestive function, immune regulation, metabolic balance, and the prevention or adjunctive management of certain disorders [99]. Their effectiveness within a balanced diet depends not only on strain-specific properties but also on the overall dietary context in which they are consumed [100].

### 6.1. Definition and Principal Groups

Probiotics are defined as live microorganisms that confer health benefits when consumed in adequate amounts [101]. Their effects are strain-specific and depend on the precise taxonomic identity, genomic and functional characteristics, viability, administered dose, formulation, duration of use, and host context [102]. Therefore, genus-level terminology should be interpreted cautiously. The most extensively studied probiotic organisms include strains of *Lacticaseibacillus rhamnosus* GG and *Bifidobacterium animalis* subsp. lactis BB-12, *Lactiplantibacillus plantarum* 299v, and the yeast *Saccharomyces cerevisiae* var. *boulardii* [103]. These strains differ in their tolerance to acid and bile, adhesion properties, metabolic activity, persistence in the gastrointestinal tract, interaction with host tissues, and documented clinical outcomes [104]. Evidence obtained with one probiotic strain cannot be automatically extrapolated to other strains of the same species or genus. Accordingly, the health effects discussed in this review refer to specific documented strains whenever the original evidence permits strain identification. When the available studies report only a genus or species, the corresponding evidence is described as genus-level or species-level and is not interpreted as demonstrating a universal effect for all members of that taxonomic group.

### 6.2. Role in Gut Health

One of the main functions of probiotics is the maintenance or restoration of the balance of intestinal microbes [97,105]. Probiotics can compete with pathogenic micro-organisms for adhesion sites and nutrients, produce antimicrobial compounds, and help create a gut environment less favorable to harmful species [106,107]. These mechanisms could help stabilize the intestinal ecosystem during dietary changes, infections, antibiotic treatment, and gastrointestinal stress, and improve the integrity of the intestinal barrier by modulating tight junction proteins, mucin production, and epithelial resilience [108]. Additionally, probiotics can modulate local immune responses in gut-associated lymphoid tissue, thereby affecting the balance between tolerance and inflammation [109]. Some specific probiotic strains have been investigated for gastrointestinal outcomes, including the management of antibiotic-associated diarrhea, lactose digestion, and selected symptoms of functional gastrointestinal disorders. For example, findings reported for *L. rhamnosus* GG or *B. animalis* subsp. *lactis* BB-12 should be attributed to those documented strains and to the conditions under which they were studied, rather than generalized to all Lacticaseibacillus or Bifidobacterium strains [41]. Other strains have been evaluated for possible effects on immune responses, inflammation, metabolic markers, or intestinal barrier function [40,110]. However, these outcomes remain dependent on the strain, dose, preparation, population, and clinical endpoint [9]. Thus, probiotic supplementation should be considered strain-specific and outcome-specific, rather than assumed to provide a uniform benefit across a genus or species.

### 6.3. Probiotics and the Health of the Whole Body

The most common discussion of probiotics centers on gastrointestinal outcomes, but their effects may extend beyond the gut. Some strains are effective in relieving symptoms of irritable bowel syndrome, improving lactose digestion, and reducing antibiotic-associated diarrhea [14,111]. Others have been investigated for potential immune support, regulation of inflammation, and treatment of metabolic diseases, such as obesity, insulin resistance, and dyslipidemia [14,112]. These broader effects are thought to be mediated by mechanisms that include gut barrier enhancement, microbial metabolite production, immune signaling, and modulation of inflammatory mediators [113]. However, the evidence is inconsistent across all conditions and probiotic preparations [114]. Therefore, probiotic supplementation should be considered strain-specific and outcome-specific rather than assumed to confer universal benefit [113]. Although selected probiotic strains have been investigated as adjuncts in particular gastrointestinal or metabolic conditions, evidence for one strain, dose, formulation, or clinical outcome cannot be generalized to all probiotic products. Probiotic supplementation should therefore not be presented as a universal therapeutic recommendation for diabetes, inflammatory bowel disease, obesity, or other disorders. Clinical use should be based on strain-specific evidence and professional assessment. Preclinical studies show that probiotics can influence microbial balance, epithelial integrity, and immune signaling, but strain specificity and host context limit direct generalization to human outcomes. Table 5 presents commonly studied probiotic genera together with representative strains for which specific evidence has been reported. The table distinguishes strain-level evidence from genus- or species-level descriptions. Where a study did not identify the strain, the evidence is labeled as genus-level or species-level. It should not be interpreted as demonstrating equivalent effects for all members of that taxonomic group.

### 6.4. Interactions with Dietary Components

The diet environment has a significant impact on the activity and viability of probiotics [110]. Some oligosaccharides and DFs are fermentable substrates that can serve as carbon sources, supporting probiotic persistence and metabolic activity [115]. This interaction underlies the notion of synbiotics, the combination of probiotics and prebiotic substrates to enhance efficacy [114]. Whole DFs and phytochemicals could be especially useful in this regard [110]. Furthermore, the overall dietary pattern, especially the quality of fats and fiber intake, may affect the efficacy of probiotic activity in the gut [87].

### 6.5. Food Matrices as Probiotic Delivery Systems

Probiotics are generally provided through fermented dairy products such as yogurt, kefir, and cultured milk, although non-dairy options are attracting increasing attention [116]. Their stability, viability, and consumer acceptance are being studied in fruit- and cereal-based matrices, plant-derived substrates, and encapsulated forms [117], with particular relevance for individuals with lactose intolerance, vegans, and those following culturally appropriate diets [118]. The food matrix could provide benefits for probiotics delivered through foods compared with supplements by improving microbial survival during storage and transit through the gastrointestinal tract [100,110]. In addition, combining probiotics with fiber- and polyphenol-rich meals may confer additive or synergistic benefits, providing a rationale for developing next-generation functional foods for gut health [118]. Probiotic studies are widely heterogeneous in terms of strains, dosages, modes of administration, and results. Many studies are small or short-term with limited surrogate endpoints. Overall, the benefits appear to be strain-specific and context-dependent, and further robust studies with unambiguous strain reporting and well-defined dietary conditions are still required.

### 6.6. Relation to Balanced Nutrition and Gut Health

Probiotics are best thought of in the wider dietary context, rather than as a stand-alone intervention [119]. Dietary environment, particularly fermentable fiber, impacts probiotic persistence and activity [120]. Thus, probiotics are interacting components of a nutrition–microbiome–health system, especially when combined with fiber-rich foods such as dates and dietary patterns that promote an appropriate ω-3/ω-6 balance [33,121,122]. These interactions may boost SCFAs synthesis, reinforce barrier function, and modify inflammatory and metabolic responses. However, the research is limited by strain specificity, variability in viability and dose, brief intervention durations, and dependence on surrogate outcomes. The modest, context-dependent confidence in the findings is further constrained by inconsistent strain-identity reporting, product stability, microbiome techniques, background diet, and low population diversity [103,121].

### 6.7. Oxidative Stress and Redox Modulation

Probiotics may affect intestinal redox balance through strain-specific metabolite production, modulation of microbial ecology, reinforcement of the epithelial barrier, and regulation of mucosal immune responses. Some probiotic strains may produce antioxidant molecules, increase the availability of reducing equivalents, or stimulate host antioxidant defenses. Other strains may reduce oxidative stress indirectly by limiting pathogen overgrowth, decreasing inflammatory signaling, and improving epithelial resistance to luminal stress [16,108,113].

The interaction between probiotics and dietary substrates is particularly relevant. Fermentable fiber and polyphenol-rich foods may support probiotic survival and microbial cross-feeding, potentially increasing the production of SCFAs and other metabolites that influence epithelial redox signaling [95]. This interaction may reduce oxidative stress by improving barrier integrity and limiting the translocation of microbial products that activate inflammatory and oxidative pathways [69]. However, these effects should not be generalized to all probiotic products, as antioxidant and redox-related activities are strain-specific and depend on the viable dose, delivery matrix, storage stability, and host conditions. Preclinical studies provide evidence that selected strains can reduce markers of lipid peroxidation, ROS generation, inflammatory signaling, or tissue oxidative injury. Human studies are less consistent and often use systemic surrogate markers rather than direct measurements of intestinal redox status [55,58,123]. Furthermore, many clinical studies do not report strain identity, viable cell counts at the time of administration, or the background diet sufficiently to permit mechanistic interpretation. The current evidence therefore supports probiotics as potential modulators of gut redox balance, but not as universally effective antioxidant interventions.

### 6.8. Link to the Mechanistic Framework

According to Figure 1, probiotics may influence gut health by modulating resident microbiota, microbial metabolites, epithelial barrier function, inflammatory signaling, and redox balance. Selected strains may stimulate host antioxidant defenses or indirectly reduce oxidative stress by limiting pathogen-associated inflammation and improving epithelial resilience. Their effects may be enhanced by fermentable substrates such as DFs and polyphenols, although human evidence for consistent changes in oxidative-stress markers remains limited and strain-specific. Future studies should report strain identity, viable dose, delivery matrix, adherence, baseline microbiota, and standardized oxidative, inflammatory, barrier, and metabolomic outcomes.

### 6.9. Evidence Quality and Challenges

Human evidence for this component is largely based on small, short-term trials and a limited number of observational studies, with most outcomes restricted to surrogate markers (e.g., glycemia, lipid profiles, systemic antioxidant or inflammatory biomarkers) rather than hard clinical endpoints. In particular, studies using whole foods and those using supplements or isolated extracts were synthesized and discussed separately, given differences in dose standardization, food matrix effects, and translational relevance to usual dietary intake. Reported findings are heterogeneous and occasionally conflicting, reflecting variation in intervention characteristics (e.g., cultivar, ripening stage, and processing for dates; fatty-acid type, dose, oxidation status, and background diet for ω-3/ω-6; fiber type, dose, solubility, fermentability, and food matrix for DFs, and strain identity, viable dose, formulation, delivery matrix, and host factors for probiotics). In contrast, animal and in vitro studies provide strong mechanistic plausibility for effects on gut microbiota composition and activity, microbial production of SCFAs, intestinal barrier integrity, oxidative stress, and inflammation. However, these preclinical models frequently employ concentrated extracts, isolated compounds, or supraphysiological doses under highly controlled conditions that do not reflect usual dietary intake or real-world consumption patterns. Consequently, while pathway-level effects are biologically plausible, they should not be equated with clinically meaningful benefits in humans. Overall, available human data support the tolerability of moderate intake and suggest selected metabolic or bowel-related benefits, but consistent, reproducible effects on gut microbiota function, fecal SCFA concentrations, validated measures of intestinal permeability, and intestinal redox status remain insufficiently demonstrated. Larger, longer-duration, well-controlled human trials using standardized, well-characterized preparations and clinically relevant endpoints are needed to clarify the translational relevance of these findings.

## 7. Integrated and Comparative Synthesis of Dates, ω-3/ω-6 Fatty Acids, DFs, and Probiotics

Dates, DFs, probiotics, and ω-3/ω-6 fatty acids are not interchangeable; each plays a distinct role in the diet–microbiota–host axis. Dates provide a whole-food matrix of fermentable carbohydrates, fiber, polyphenols, and micronutrients; fiber serves as the primary substrate for microbial fermentation; probiotics deliver specific live strains; and ω-3/ω-6 fatty acids modulate membrane composition, lipid mediators, and immune signaling, thereby indirectly shaping the gut environment. Their combined value, therefore, rests on mechanistic complementarity rather than proven interaction. Evidence indicates convergence on microbiota activity, SCFA production, barrier integrity, oxidative stress, and inflammation, but the strength and directness of support differ markedly across components and outcomes.

**Figure 2:**
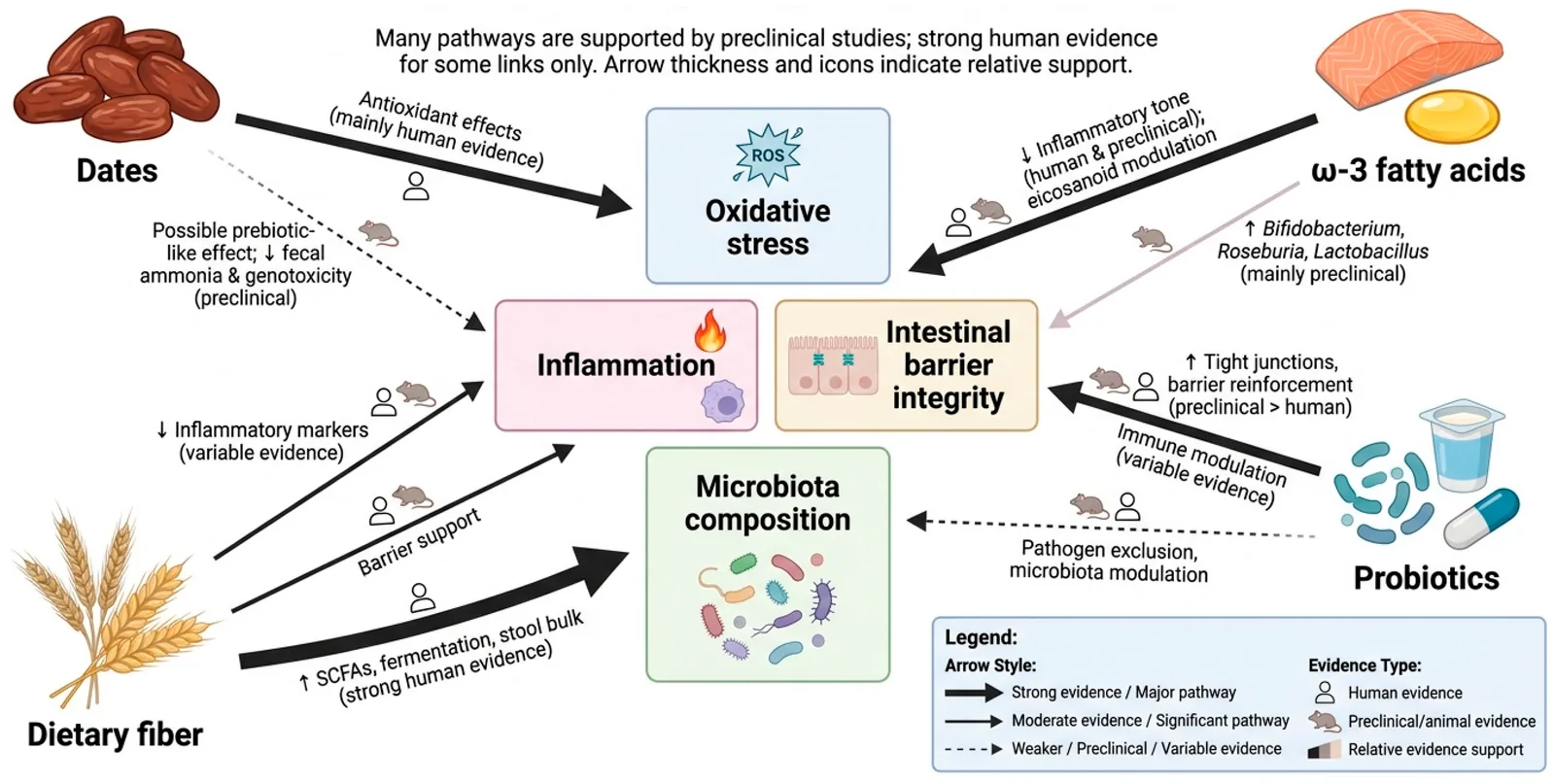
Evidence summary of the effects of dates, ω-3/ω-6 fatty acids, DFs, and probiotics on microbiota composition and function, SCFA production, intestinal barrier integrity, oxidative stress, redox balance, and inflammation. The figure distinguishes between relatively strong evidence for fiber-related fermentation and selected ω-3 inflammatory outcomes, and emerging or limited evidence for direct human changes in intestinal oxidative stress, barrier function, and combined-component effects.

The nutritional importance of dates, ω-3 and ω-6, DFs, and probiotics is more persuasive when these components are considered as part of an interacting dietary system rather than as isolated factors, Figure 2 [124,125]. DFs and polyphenols may modulate the gut microbiota, enhance SCFA production, and strengthen barrier function, indicating that combinations of bioactive food constituents may have effects beyond those of individual components alone [10,33]. At the same time, ω-3 has been associated with microbiome-related and anti-inflammatory effects, including enrichment of some beneficial bacterial groups in some intervention studies, which supports the idea that fat quality may affect the same physiological pathways targeted by fiber- and pro-biotic-based strategies [39,70].

As shown in Figure 2, oxidative stress represents a point of convergence but not an identical mechanism across the four components. Dates may provide phenolic antioxidant activity; fiber may influence redox balance through fermentation and metabolic regulation; probiotics may act through strain-specific microbial and host pathways; and ω-3/ω-6 fatty acids may alter membrane oxidation and lipid mediator formation. The level of human evidence remains uneven across these mechanisms.

### 7.1. Shared Mechanistic Pathways

#### 7.1.1. Microbiota Composition and Function

DFs have the most direct nutritional relationship with the gut microbiota because fermentable fibers provide substrates for microbial growth and metabolism [18,91]. Their effects, however, depend on chemical structure, viscosity, fermentability, dose, habitual diet, and the baseline microbiome [76,87]. Dates may contribute through both fiber and polyphenols. Unlike purified fiber, dates deliver these constituents within a whole-food matrix; intestinal microorganisms may transform date-associated polyphenols into biologically active metabolites [10,33,103]. Human evidence indicates that date consumption can improve bowel-related outcomes and selected fecal markers even when broad changes in microbiota composition are not detected [125,126]. This distinction suggests that functional microbial activity may be more informative than taxonomic abundance alone.

Probiotics act through a different route. They introduce defined microorganisms that may compete with pathogens, produce antimicrobial substances, alter luminal metabolites, and interact with epithelial and immune cells [97,105]. Their effects are highly strain-specific and do not necessarily involve durable colonization or large shifts in the resident microbiota [102]. Fermentable fiber may improve the ecological conditions for probiotic activity, which provides the rationale for synbiotic combinations [85,114]. Nevertheless, the presence of fiber does not guarantee probiotic efficacy; compatibility between the strain, substrate, dose, food matrix, and host microbiome must be demonstrated experimentally [110,120].

The effect of ω-3/ω-6 fatty acids on the microbiota is less direct and more context-dependent. ω-3 fatty acids may influence microbial ecology by altering bile acid metabolism, intestinal inflammation, mucosal lipid composition, and host-derived substrates [62,70]. Some human and preclinical studies report changes in selected taxa or in microbial diversity after ω-3 intake, but the findings are inconsistent [39,127]. For ω-6 fatty acids, effects cannot be inferred solely from the amount consumed because linoleic acid and arachidonic acid have physiological functions, and their impact depends on dose, source, tissue incorporation, concurrent ω-3 intake, and the overall dietary pattern [63,64]. Thus, fatty acids appear to modify the ecological and immunological context in which fiber, dates, and probiotics operate rather than serving as primary fermentable substrates.

#### 7.1.2. SCFA Production and Microbial Metabolites

The fiber–microbiota–SCFA axis is the most clearly established mechanistic connection among the four components. Colonic fermentation of suitable fibers produces acetate, propionate, and butyrate, which can support colonocyte energy metabolism, epithelial integrity, immune regulation, and systemic metabolic signaling [40]. Dates may contribute to this axis through their fiber and fermentable carbohydrate fractions, although the amounts and fermentability of these components vary among cultivars, ripening stages, and processing methods [6,25]. Polyphenol metabolism may generate additional microbial products, but the quantitative and clinical relevance of these metabolites remains incompletely characterized [128,129].

Probiotics may contribute to SCFA-related effects directly or indirectly, depending on the strain’s metabolic capabilities and the available substrate [14,112]. In many cases, their most plausible role is cross-feeding: probiotic organisms or resident bacteria may transform substrates into intermediates that are subsequently used by other SCFA-producing microorganisms [130,131]. This mechanism is biologically plausible but requires strain-level and metabolomic confirmation. ω-3/ω-6 fatty acids may influence SCFA production indirectly by modifying the intestinal environment and inflammatory tone; direct human evidence of a consistent increase in SCFAs after fatty acid intervention remains limited [69]. Accordingly, a combination of dates or other fiber-rich foods with a compatible probiotic could reasonably be hypothesized to affect microbial metabolites more strongly than either component alone [33]. However, this remains a testable hypothesis rather than an established clinical conclusion. Future studies should quantify fecal and, where feasible, circulating SCFAs, along with microbial gene pathways, rather than report taxonomic changes without functional measurements.

#### 7.1.3. Intestinal Barrier Integrity and Immune Signaling

All four components may influence the intestinal barrier, but through different proximal mechanisms. SCFAs generated from fermentable fiber can support epithelial energy metabolism and tight-junction regulation [40]. Date polyphenols may reduce oxidative and inflammatory pressure on epithelial cells, although much of this evidence is derived from experimental models or concentrated extracts [35]. Probiotics may interact directly with epithelial cells, mucins, tight junction proteins, and mucosal immune pathways; nevertheless, their effects vary considerably among strains and clinical indications [108,109]. ω-3 fatty acids may alter membrane composition and inflammatory lipid mediators, thereby influencing mucosal immune responses and barrier function [68].

These mechanisms could be complementary: fiber may provide microbial substrates, probiotics may contribute strain-specific epithelial or immunological effects, dates may supply polyphenols within a fermentable food matrix, and ω-3-rich foods may help shape inflammatory tone [10,132]. Yet evidence for additive or synergistic improvements in intestinal permeability remains insufficient, as few human studies examine these components in combination or use harmonized permeability endpoints [133,134]. Claims concerning barrier improvement should therefore remain cautious unless supported by validated human measures, such as standardized permeability tests or clinically relevant mucosal outcomes.

#### 7.1.4. Oxidative Stress and Inflammation

Oxidative stress and inflammation represent shared downstream domains, but the evidence pathways are not equivalent. Date phenolics have antioxidant and anti-inflammatory potential in biochemical, cellular, and animal models, whereas human studies have primarily assessed circulating surrogate biomarkers, yielding variable findings [26,27]. Fiber may indirectly reduce the inflammatory burden through improved microbial fermentation, SCFA signaling, glycemic regulation, and body weight control [45,87]. Probiotics may modulate inflammatory responses through microbial products, epithelial signaling, and immune regulation, but results are indication- and strain-specific [14,112].

ω-3 fatty acids have the most direct biochemical connection to the resolution of inflammatory mediators because they alter membrane lipid composition and serve as precursors for specialized pro-resolving mediators [53,58]. Human evidence is stronger for selected systemic and cardiometabolic outcomes than for gut-specific oxidative-stress or inflammation endpoints [59,73]. ω-6 fatty acids should not be categorized as inherently harmful: they are physiologically essential, and their effects depend on their specific identity, dose, tissue context, and balance with ω-3 fatty acids [63,64]. The relevant dietary objective is therefore improvement of overall fat quality and pattern, not elimination of ω-6 fatty acids. The shared implication is that antioxidant and anti-inflammatory effects may emerge from coordinated changes in substrate supply, microbial metabolism, epithelial protection, and host lipid signaling [10,135]. At present, the literature supports mechanistic convergence more strongly than it demonstrates a clinical interaction among all four components.

### 7.2. Relative Evidence Strength Across Components

The evidence is strongest when the outcome is closely related to the component’s primary biological action and weaker when several unmeasured steps separate exposure from the outcome, Table 6. Fiber has relatively consistent human support for bowel function and selected metabolic outcomes, with moderate-to-strong evidence for fermentation and SCFA-related mechanisms [45]. Probiotics have moderate evidence for selected gastrointestinal indications, but effects cannot be generalized across strains, doses, formulations, or diseases [14,103]. Dates have promising human evidence for tolerability, bowel-related outcomes, glycemic responses, and selected oxidative and inflammatory markers. Still, direct evidence for reproducible changes in microbiota, SCFAs, and barriers remains limited [26,33]. ω-3/ω-6 research is extensive, though it is weighted toward cardiometabolic and systemic outcomes; gut-specific effects are moderate or limited and strongly dependent on the type of fatty acid and dietary context [58,62].

At the combination level, direct human evidence is particularly sparse. Fiber–probiotic combinations have the strongest translational rationale because the interaction is conceptually synbiotic and can be evaluated with defined substrates and strains [114,129,136]. Dates–fiber represents a whole-food relationship, but the incremental effect of date-specific polyphenols beyond total fiber intake is not established [6,33]. Dates–probiotic and dates–ω-3 combinations remain plausible functional-food strategies, yet direct controlled human evidence is limited [121,137]. ω-3–fiber and ω-3–probiotic combinations have complementary mechanistic support, but most available findings are indirect or derived from interventions that do not isolate interaction effects [70,138], as illustrated in Table 7.

### 7.3. Translational Implications and Research Gaps

The most defensible translational implication is not that all four components should be prescribed as a universal therapeutic combination. Rather, a food-based dietary pattern can be designed to include culturally acceptable sources of dates, fermentable fiber, ω-3-rich foods, and validated probiotic foods or preparations, while maintaining appropriate portion sizes and overall energy balance [121,139]. Dates may be especially useful as a culturally relevant whole-food carrier of fiber and polyphenols, but their natural sugar and energy density should be considered [6,37]. Probiotic products should be selected according to documented strain identity, viable dose at the end of shelf life, delivery matrix, and the intended health outcome [102,103]. ω-3/ω-6 recommendations should focus on food quality and replacement of less favorable dietary fats rather than on an isolated ratio target [52,65].

A practical research model would compare: (1) usual diet; (2) dates or date-derived food; (3) defined fiber; (4) probiotic preparation; and (5) factorial combinations, with ω-3 enrichment or improved fat quality incorporated as an additional factor. Trials should use realistic food portions and clinically meaningful durations rather than relying exclusively on concentrated extracts or high-dose supplements [140,141]. Primary outcomes should include bowel function, glycemic control, lipid profile, and quality of life. In contrast, mechanistic outcomes should include microbiota function, fecal and circulating SCFAs, bile acids, validated measures of intestinal permeability, inflammatory mediators, and oxidative stress biomarkers [126,142].

Several methodological gaps currently limit synthesis across studies:

- Food and intervention identity are inconsistently reported. Studies should specify the date, cultivar, ripening stage, processing, polyphenol and fiber content, fatty acid composition, probiotic strain, viable dose, and storage stability [6,24,103].
- Background diet is often inadequately controlled. Total fiber, fermented-food intake, energy intake, medication use, and concurrent fatty-acid supplements can modify outcomes [143,144].
- Micobiome endpoints are frequently taxonomic rather than functional. Metagenomics, metatranscriptomics, metabolomics, and SCFA measurements should complement 16S rRNA profiling [145,146].
- Barrier and oxidative-stress outcomes lack harmonization. Studies should prespecify validated permeability tests and a justified panel of oxidative and inflammatory biomarkers [147,148].
- Interindividual response is insufficiently addressed. Baseline microbiota, metabolic phenotype, habitual diet, age, sex, medication, and geographic or cultural dietary context may modify treatment response [149,150].
- Interaction is often inferred rather than tested. Factorial designs, mediation analyses, and dose–response models are needed to distinguish additive, synergistic, and independent effects [131,151].

Overall, the unique contribution of this review is the separation of pathway-level plausibility from human translational evidence. Dates, DFs, probiotics, and ω-3/ω-6 fatty acids converge on related biological domains, but they do not have equal evidentiary status or identical mechanisms. Fiber currently provides the clearest microbiota–SCFA link; probiotics provide selected strain-dependent gastrointestinal effects; dates offer a culturally relevant whole-food matrix with promising but incompletely characterized microbial and antioxidant actions; and ω-3/ω-6 fatty acids primarily shape host lipid signaling and inflammatory context. The proposed integrative model is therefore best viewed as a framework for designing and testing food-based combinations, not as proof that the four components already constitute a clinically validated synergistic intervention.

## 8. Applications in Diet and Nutrition

These findings support food-based dietary patterns centered on whole foods, high-quality fats, fiber, and fermented foods [121]. Integrating dates, ω-3-rich foods, fiber-rich plant sources, and probiotic-containing products aligns with current dietary guidance that emphasizes nutrient interactions over isolated intake [152,153]. Such patterns are particularly relevant for gut health, as they may enhance microbial diversity and reduce inflammatory burden [10].

### 8.1. Practical Recommendations for Daily Diets

A practical dietary strategy should begin with the regular inclusion of whole, minimally processed foods that naturally provide the nutrients and bioactives discussed in this review [154]. Dates can be incorporated in moderate amounts as part of snacks, breakfast meals, or mixed dishes, especially when paired with protein- or fat-containing foods, such as nuts or fermented dairy, to improve satiety and moderate the glycemic response [84,122]. At the same time, increasing intake of ω-3-rich foods such as fatty fish, walnuts, chia seeds, flaxseed, and selected fortified foods may help improve the ω-3/ω-6 balance in the overall diet [139,155]. Practical meal planning should combine dates, fiber-rich foods, healthy fats, and probiotic foods in moderate portions [152,156]. This strategy is preferable because whole foods provide fiber alongside vitamins, minerals, and phytochemicals that may act synergistically in the gastrointestinal tract [157].

### 8.2. Food Combinations and Meal Planning

Dates can be mixed with almonds for a balanced snack [158]. At the same time, diets that combine fatty fish, legumes, vegetables, whole grains, and fermented dairy provide complementary supplies of beneficial fats, fiber, and microbial substrates [159]. Such combinations may increase nutritional density and lead to more beneficial metabolic and gastrointestinal responses when fermentable substrates, unsaturated fats, and bioactive substances are combined [160,161]. The meal planning technique should also include portion size, calorie density, and the overall nutritional context. Probiotic foods should also be a complement, not a replacement for, the typical consumption of fiber-rich meals and healthy fats, since the microbial benefits depend on the dietary matrix in which they are ingested [96].

### 8.3. Public Health and Preventive Nutrition

From a public health perspective, these dietary components are important as they simultaneously address multiple common nutritional deficits and risks for chronic diseases [43]. Many communities consume insufficient amounts of DFs and foods high in ω-3 fatty acids, instead consuming refined, ultra-processed foods, which may compromise microbial and metabolic health. Increased intake of fiber-rich plant foods, fermented foods, and healthy fats may help reduce the risk of obesity, type 2 diabetes, cardiovascular disease, and gut-related illnesses [42,162]. This method is also in line with culturally adjusted dietary counseling [163]. In areas where dates are a traditional part of the diet, they might be a realistic starting point for encouraging healthy snack behaviors and whole-food alternatives [125]. They can be mixed with nuts, probiotic foods, or other fiber-rich foods and incorporated into dietary models that reflect low-calorie food culture while remaining compatible with current preventive nutrition goals [145].

### 8.4. Implications for Dietary Recommendations and Functional Foods

The inclusion of dates, healthy fats, DFs, and probiotics aligns with the broader trend in modern dietary guidelines that increasingly emphasize whole foods, food quality, and gut-friendly dietary patterns [153]. This viewpoint provides a strong basis for the inclusion of these components in evidence-based dietary counselling and public health messaging [158]. There is also potential for the food industry and applied nutrition industries to produce functional goods that integrate these in acceptable, scientifically supported ways [121]. Examples include date-based snacks supplemented with nuts and seeds; probiotic and fiber dairy or non-dairy products; and formulations designed to increase ω-3 consumption in habitual diets [49,151]. Such improvements may be particularly effective if they retain the integrity of whole foods, enhance consumer adherence, and are designed with local preferences and metabolic health outcomes in mind [121].

## 9. Safety and Tolerability Considerations

Although dates, DFs, probiotics, and ω-3/ω-6 fatty acids are common in the diet, their safety and tolerability warrant attention, especially with rapid increases in intake or supplement use. Rapidly increasing fermentable fiber (from dates, supplements, or high-fiber diets) can cause bloating, flatulence, abdominal discomfort, and altered stool consistency, particularly in individuals with functional gastrointestinal disorders or low habitual fiber intake; gradual titration and adequate fluids are recommended. Probiotics are generally well tolerated, but rare bloodstream infections and fungemia have been reported in critically ill, immunocompromised, or severely debilitated patients (e.g., those with central venous catheters or short bowel syndrome), warranting caution and medical supervision. Allergic reactions to probiotic products or excipients, though uncommon, may occur in individuals with allergies to dairy, soy, or other formulation components. Dates are energy-dense and sugar-rich; excessive intake may promote positive energy balance and weight gain and should be accounted for within total energy and carbohydrate targets, especially in obesity, insulin resistance, or diabetes. High-dose ω-3 supplements may increase bleeding risk, particularly with anticoagulant/antiplatelet therapy, and have been linked to dose-dependent atrial fibrillation risk in some trials; fish or algae oil supplements may also cause gastrointestinal side effects and, depending on quality, exposure to oxidized lipids or contaminants. These components should be viewed as part of a balanced diet; when used as supplements or at high doses, intake should be individualized and, when appropriate, supervised by a qualified healthcare professional.

## 10. Geographic and Cultural Applicability

The applicability of these findings varies across regions and dietary cultures. In the Middle East and North Africa, dates are culturally embedded and can meaningfully contribute to fiber, micronutrient, and polyphenol intake within familiar dietary patterns; elsewhere, where dates are occasional snacks or sweeteners, their impact on diet quality and gut outcomes is likely smaller and more dependent on portion size and frequency. ω-3 intake patterns also differ, from high fish consumption in coastal, Northern European, and East Asian diets to greater reliance on plant sources (flaxseed, chia, walnuts) elsewhere, influencing baseline fatty-acid status and the potential effect of modifying the ω-3/ω-6 balance. Fiber sources vary from cereal-dominant to legume-, fruit-, vegetable-, or tuber-based diets, with some populations shifting toward lower-fiber, processed diets. Probiotic exposure is similarly heterogeneous, shaped by fermented food traditions (yogurt, kefir, kimchi, miso), regulations, and supplement access. Most mechanistic and clinical studies originate from limited geographic areas and homogeneous populations, limiting generalizability. Future research should prioritize diverse populations, culturally relevant dietary patterns, and locally available foods to clarify regional differences and support context-specific nutrition recommendations.

## 11. Concluding Remarks and Future Research Directions

Future research should move beyond isolated effects of dates, ω-3/ω-6, DFs, and probiotics to evaluate their interactions within dietary patterns and microbiome-focused interventions. The gut microbiome is increasingly recognized as a functional interface that links food to metabolic and immunological consequences, facilitating the integration of dietary exposures with microbiome and metabolomics investigations [134]. This is particularly important for functional foods because efficacy relies on constituent qualities, the host, the microbial milieu, and the food matrix.

### 11.1. Gaps in Current Knowledge

A key limitation in current research is the lack of well-designed human trials that assess these components in combination rather than in isolation, thereby limiting understanding of potential additive or collaborative effects [136,147]. Outcomes are also affected by changes in date cultivar, processing, probiotic strains, fiber types, and sources of fatty acids [33,140]. In addition, the use of broad clinical indicators and inadequate characterization of microbial activity and host-microbiome interactions limits interpretation [146]. Metabolomics and functional microbial profiling are emerging techniques that may better reflect diet-related responses than taxonomic metrics alone [164].

### 11.2. Future Studies’ Fields

Future research should focus on randomized controlled trials on realistic food-based combinations, such as date-rich fiber matrices with probiotics or ω 3-enriched diets with prebiotic-rich foods [165]. Studies should be of standardized design, with unambiguous characterization of the therapies, and evaluate clinically important outcomes such as glycemic control, lipid profiles, inflammation, bowel function, and quality of life [140,141]. Mechanistic investigation is needed to elucidate the involvement of SCFA synthesis, barrier function, bile acid metabolism, immunological modulation, and microbial cross-feeding [124,133]. Attention should also be paid to dose–response relationships, including recommended consumption ranges across groups and variation according to microbiota composition, metabolic status, and habitual diet [149]. Probiotic effectiveness is also strain-specific and viability-dependent, yet this remains reported erratically [123].

### 11.3. Research on Nutrition and Gut Health: Emerging Trends

A key emerging direction is nutrition guided by the gut microbiome, host phenotype, and metabolite profiles [166]. Multi-omics approaches, including microbiomics, metabolomics, transcriptomics, and epigenetics, are increasingly used to explain interindividual variability in dietary responses and to identify subgroups most likely to benefit from specific combinations of fiber, probiotics, polyphenols, and healthy fats [150,167]. Concurrently, microbiome-targeted strategies are expanding beyond probiotics and prebiotics to include synbiotics, postbiotics, and fermented functional systems [41,168]. Recent evidence indicates that fermented foods and targeted formulations may influence systemic health through live microbes, bioactive metabolites, modified polyphenols, and enhanced fiber fermentability [119,168]. These advances support the development of next-generation functional foods incorporating date-derived ingredients, tailored fatty acids, and probiotic cultures to target specific health outcomes [169].

### 11.4. Translational and Methodological Considerations

Future research will have translational value only if it is standardized, reproducible, and applicable in real-world settings [170]. In particular, there is a need for more harmonization in the reporting of food composition, cultivar or strain identity, processing, dose, duration, adherence, and background diet to permit comparison across research [144]. Longitudinal designs with frequent biological sampling and established nutrition assessment techniques are particularly important to identify transitory from persistent effects [148]. Artificial intelligence and machine learning may aid in synbiotic design and in predicting response, but should be used to supplement mechanistic and clinical validation [171,172]. To summarize, a shift toward mechanism-based, personalized, and clinically relevant nutrition research, including whole foods, microbiome dynamics, metabolic biomarkers, and human outcomes, will help provide evidence for the roles of fats, fatty acids, DFs, and probiotics as coordinated components of gut health strategies.

## 12. Limitations and Heterogeneity of the Evidence

Interpretation is limited by substantial heterogeneity in study designs, populations, interventions, outcomes, and reporting. Human studies, while most relevant for tolerability and associations with bowel, metabolic, inflammatory, and oxidative-stress outcomes, are often small, short-term, and reliant on surrogate markers. Animal and in vitro studies provide valuable mechanistic insights but cannot be assumed to predict clinically meaningful effects in humans. Considerable heterogeneity exists among the interventions. Date studies differ in cultivar, ripening stage, preparation, processing conditions, portion size, and duration, which can alter fiber, sugars, phenolics, minerals, and other bioactives [6,24,25]. DFs’ studies vary in type, solubility, viscosity, fermentability, structure, food matrix, dose, and administration, limiting extrapolation from isolated fibers to whole-food sources [77,78,87]. Fatty-acid interventions differ in type, source, dose, oxidation, tissue incorporation, total fat intake, and background ω-3/ω-6 pattern, complicating interpretation of inflammation, oxidative stress, membrane signaling, microbiota, and barrier outcomes [52,53,64]. Probiotic efficacy is strain-specific and depends on identity, viable dose, formulation, delivery matrix, stability, duration, adherence, and clinical condition, precluding generalization across strains or products [101,102,103].

Study populations vary in age, sex, health status, metabolic phenotype, habitual diet, medication use, geography, and baseline microbiota, which may influence fermentation, SCFA production, probiotic persistence, and redox responses. Limited demographic diversity and inconsistent reporting reduce external validity [168,171]. Outcome heterogeneity further limits comparability, with studies assessing microbial abundance, diversity, SCFAs, bowel frequency, permeability, cytokines, antioxidant capacity, lipid peroxidation, glutathione, and metabolic biomarkers. Microbiota outcomes are often taxonomic rather than functional, and oxidative-stress findings may rely on systemic surrogates or chemical assays rather than direct intestinal measurements [69,123,166].

This review did not perform a meta-analysis because studies differed markedly in populations, interventions, comparators, outcomes, follow-up, and quality, and the evidence ratings (“strong,” “moderate,” “limited,” “emerging”) are qualitative judgments rather than formal certainty estimates. Publication bias, selective reporting, language restrictions, and incomplete study capture may influence the synthesis, and conclusions about combined effects must be clearly separated from those about individual components. Human data support moderate dietary intake, selected fiber benefits, strain-specific probiotic effects, and certain systemic ω-3 outcomes. Still, consistent improvements in microbiota function, SCFAs, permeability, oxidative stress, or redox balance have not yet been firmly demonstrated, with animal and in vitro work mainly providing mechanistic plausibility at non-habitual doses. Critically, no robust human trials have tested a standardized combination of dates, defined fiber, controlled ω-3/ω-6 intake, and characterized probiotics with comprehensive gut and redox endpoints, so proposed additive or interactive effects remain hypothesis-generating, underscoring the need for randomized factorial studies with standardized preparations, controlled diets, validated barrier/redox measures, and integrated microbiome–metabolomic analyses.

## 13. Conclusions

This review indicates that dates, ω-3 and ω-6 fatty acids, DFs, and probiotics are complementary dietary components with potential relevance to gut health, inflammatory regulation, and metabolic homeostasis. Dates provide fiber, phenolic compounds, and minerals that may support antioxidant capacity, intestinal function, and microbial activity. ω-3 and ω-6 fatty acids influence membrane structure, lipid-mediator production, and cardiometabolic pathways, although their effects depend on dietary balance and context. DFs support gastrointestinal function and microbial fermentation, including SCFA production, whereas probiotics may exert strain-specific effects on microbial balance, barrier function, and immune responses. However, the available evidence is heterogeneous and is stronger for individual components and mechanistic pathways than for combined interventions or clinically meaningful disease outcomes. Many findings derive from preclinical studies, surrogate biomarkers, or short-term human trials with variable preparations, doses, and populations. Therefore, the potential collaborative effects of these components and their role in disease prevention should be considered hypothesis-generating rather than established. Future adequately powered human intervention studies using standardized preparations, realistic intake levels, and clinically relevant outcomes are needed to clarify their combined effects and translational value.

## Figures and Tables

**Table 1:** Nutritional and bioactive profiles of dates.

Component	Major Constituents	Functional Relevance	Notes on Variability
Macronutrients	Carbohydrates, proteins, fats	Energy provision, satiety, dietary quality	Depends on cultivar and stage of ripeness
DFs	Insoluble and soluble fiber	Bowel regularity, glycemic modulation, microbiota support	Varies with variety and processing methods
Micronutrients	Potassium, magnesium, calcium, phosphorus, iron, B vitamins	Electrolyte balance, metabolism, antioxidant support	Affected by climatic conditions and cultivation practices
Phenolic compounds	Phenolic acids, flavonoids, tannins	Antioxidant and anti-inflammatory activity	Highly dependent on cultivar and stage of maturity
Other bioactives	Carotenoids, tocopherols, phytosterols	Redox balance, membrane protection	Influenced by storage conditions and postharvest handling

**Table 2:** Summary of health effects of date consumption in human and experimental studies, including major findings and key methodological limitations.

Study Type	Population/Model	Intervention/Exposure	Main Outcomes Assessed	Key Findings	Main Limitations
Human clinical studies	Healthy adults, metabolic disorders	Whole dates or date servings	Glycemia, lipids, antioxidant markers, bowel function	Generally well tolerated; potential metabolic and gut benefits	Low sample sizes; short study duration; variable date cultivars and doses; outcomes are mostly surrogate metabolic and gut markers rather than hard clinical endpoints
Animal studies	Rodent models	Date extracts or whole-date preparations	Oxidative stress, inflammation, tissue protection	Support mechanistic plausibility	Often use extracts or high doses; controlled experimental conditions; limited extrapolation to usual human dietary intake
In vitro studies	Cell or biochemical models	Isolated compounds or extracts	Antioxidant and anti-inflammatory activity	Bioactive fractions show protective effects	Use isolated compounds without the food matrix; artificial conditions; focus on biochemical endpoints with limited translational relevance

**Table 3:** ω-3 and ω-6: classification, sources, and key health implications.

Fatty Acid	Family	Main Dietary Sources	Major Physiological Roles	Potential Health Relevance	Key Limitations/Notes
α-linolenic acid	ω-3	Flaxseed, chia, walnuts	Essential precursor fatty acid	Supports ω-3 intake balance	Limited conversion to EPA and DHA in humans; effects depend on background diet and total fat quality
Eicosapentaenoic acid (EPA)	ω-3	Fatty fish, fish oils	Eicosanoid synthesis, inflammation resolution	Cardiometabolic and inflammatory benefits	Evidence is stronger for triglyceride lowering than for microbiota or barrier outcomes; intervention doses and duration vary across studies
Docosahexaenoic acid (DHA)	ω-3	Fatty fish, algae	Membrane structure, neural function	Brain, vision, and cellular health	Gut-related outcomes are less consistently studied than systemic effects; many findings derive from surrogate biomarkers
Linoleic acid (LA)	ω-6	Vegetable oils, nuts, and seeds	Membrane function, lipid signaling	Essential, but balance with ω-3 is important	Health effects are context-dependent; it is difficult to separate LA effects from overall dietary pattern and ultra-processed food intake
Arachidonic acid (AA)	ω-6	Animal foods	Eicosanoid precursor	Physiological necessity; contextual effects depend on intake balance	Often discussed as pro-inflammatory, but effects depend on dose, metabolic context, and concurrent ω-3 intake

**Table 4:** Dietary fiber: types, sources, and mechanistic pathways.

Fiber Type	Main Characteristics	Common Food Sources	Gastrointestinal Effects	Microbiota and Systemic Effects	Key Limitations/Notes
Soluble fiber	Dissolves in water, often viscous	Fruits, oats, legumes	Slows gastric emptying and glucose absorption	Supports SCFAs production and lipid modulation	Effects vary by viscosity and fermentability; many studies use isolated supplemental fibers rather than whole foods
Insoluble fiber	Adds bulk, less fermentable	Whole grains, bran, and vegetables	Promotes stool bulk and transit	Supports bowel regularity	Less direct evidence for SCFAs-related effects; benefits often inferred from bowel outcomes rather than mechanistic endpoints
Fermentable fiber	Substrate for colonic bacteria	Legumes, fruits, selected functional ingredients	Improves colon health	Increases SCFAs and beneficial microbial activity	Heterogeneity in source, dose, and microbiome methods limits comparability across studies
Prebiotic fiber	Selectively stimulates beneficial microbes	Chicory, inulin-rich foods, and some fruits	Supports gut function	Enhances microbial balance and barrier integrity	Selectivity and efficacy depend on fiber type, host microbiota, and background diet; long-term clinical outcomes are less well established

**Table 5:** Commonly studied probiotic genera and representative strains, delivery forms, documented effects, and limitations.

Probiotic Genus and Representative Strain	Typical Delivery Form	Documented or Investigated Gut-Related Effects	Potential Systemic Effects	Evidence Level and Strain-Specific Interpretation	Key Limitations
***L. rhamnosus* GG**	Fermented dairy products, capsules, and other supplements	Investigated for intestinal barrier support, interaction with epithelial cells, and selected gastrointestinal outcomes	Immune modulation and inflammatory regulation have been investigated	Evidence should be attributed specifically to strain GG; it should not be generalized to all *L. rhamnosus* or former Lactobacillus strains	Effects depend on dose, viability, formulation, population, and clinical endpoint
***B. animalis* subsp. *lactis* BB-12 **	Yogurt, fermented dairy products, capsules, and supplements	Investigated for gastrointestinal function, microbial balance, and intestinal resilience	Potential immune effects have been reported in selected settings	Findings apply to BB-12 and cannot automatically be extrapolated to other Bifidobacterium strains	Outcomes vary according to dose, duration, background diet, host microbiota, and study design
***L. plantarum* 299v**	Capsules, functional foods, and fermented products	Investigated for intestinal comfort, barrier-related responses, and selected functional gastrointestinal outcomes	Possible effects on inflammatory and metabolic markers have been studied	Evidence is strain-specific to 299v; effects should not be attributed to all *L. plantarum* strains	Human evidence remains heterogeneous, and clinical benefits may depend on the target population and outcome measure
***S. cerevisiae* var. *boulardii***	Capsules, sachets, and oral preparations	Investigated particularly in relation to antibiotic-associated and infectious diarrhea and intestinal recovery	Possible effects on mucosal and immune responses have been reported	Evidence should be linked to the documented *S. cerevisiae* var. *boulardii* preparation and should not be generalized to all *Saccharomyces* yeasts	Preparations may differ in viability, dose, manufacturing, and clinical indication
***Lactobacillus* spp. or other lactic acid bacteria, strain not reported**	Fermented foods and supplements	General mechanisms such as acid production, competitive exclusion, and possible barrier interaction have been proposed	Possible immune or metabolic effects have been investigated	Genus-level or unspecified-strain evidence only; no specific clinical effect should be assigned to the entire genus	Lack of strain identification, variable viability and dose, small sample sizes, short follow-up, and limited comparability
***Bifidobacterium* spp., strain not reported**	Dairy products, fermented foods, and capsules	General associations with microbial balance and SCFA-related metabolism have been described	Possible gut and immune effects have been investigated	Genus-level or unspecified-strain evidence only; findings cannot be considered representative of all *Bifidobacterium* strains	Strain, dose, viability, formulation, host diet, and microbiota are frequently incompletely reported
***Lacticaseibacillus* spp., strain not reported**	Functional foods and supplements	Fermentation-related activity and possible barrier effects have been described	Metabolic and inflammatory effects have been investigated	Genus-level evidence only unless a specific strain is identified	Recent taxonomic changes and inconsistent naming complicate comparison with older studies; the evidence base is also uneven across strains

**Table 6:** Comparative evidence strength across the five mechanistic domains. Classifications are qualitative and reflect consistency, directness, and translational relevance rather than a formal meta-analytic grading system.

Component	Microbiota Composition/Function	SCFAs and Microbial Metabolites	Barrier Integrity	Oxidative Stress	Inflammation
Dates	Limited–moderate; human findings are heterogeneous and cultivar-dependent	Limited; few human studies directly measure SCFAs	Limited; mainly mechanistic, animal, or indirect human evidence	Moderate but variable; human studies commonly use surrogate biomarkers	Limited–moderate; results vary with dose, cultivar, duration, and population
ω-3/ω-6 fatty acids	Moderate for selected microbial changes; highly dependent on fatty-acid type and background diet	Limited; direct SCFA outcomes are inconsistently assessed	Limited–moderate; heterogeneous human and preclinical findings	Moderate, particularly for ω-3-related systemic outcomes; gut-specific evidence is incomplete	Moderate–strong for selected ω-3 outcomes; ω-6 effects are context-dependent
DFs	Moderate–strong; effects depend on fiber structure, dose, and baseline microbiome	Moderate–strong; most direct pathway, although responses vary among fibers and individuals	Moderate; plausible and supported by selected human measures, but endpoints are not standardized	Limited–moderate; often indirect through microbial and metabolic effects	Moderate; generally favorable but heterogeneous across fiber types and populations
Probiotics	Moderate; strain-specific and not always associated with durable colonization	Limited–moderate; dependent on strain, substrate, and cross-feeding	Moderate for selected strains and indications	Limited; consistent human evidence is insufficient	Moderate; indication-, strain-, dose-, and host-dependent

Evidence-strength classifications are qualitative and reflect the consistency, directness, and translational relevance of available human evidence. They should not be interpreted as formal meta-analytic grades. Human evidence is particularly limited for combined interventions and for simultaneous measurement of microbiota, SCFAs, barrier integrity, oxidative stress, and inflammation.

**Table 7:** Evidence grading for component combinations.

Combination	Direct Human Data	Animal or In Vitro Support	Current Interpretation
**Dates + DFs**	Limited direct human data, mainly from whole-date studies assessing bowel or metabolic outcomes	Moderate mechanistic support for fiber fermentation and polyphenol–microbiota interactions	Plausible whole-food interaction; the independent contribution of date polyphenols requires clarification
**Dates + probiotics**	No convincing direct human trials establishing a synergistic effect on gut outcomes	Mechanistic and formulation-based support for a date matrix supporting probiotic delivery or activity	Promising functional-food concept; currently preclinical or hypothesis-generating
**Dates + ω-3/ω-6**	Direct combination evidence is absent or very limited	Mechanistic support through complementary antioxidant, membrane, and inflammatory pathways	Hypothetical additive interaction requiring controlled dietary trials
**Fiber + probiotics**	Limited-to-moderate human evidence for selected synbiotic products and strains	Strong mechanistic and preclinical support for substrate availability, cross-feeding, and barrier effects	Best-supported combination concept, but efficacy remains product- and strain-specific
**ω-3 + fiber**	Limited indirect human evidence from dietary-pattern or combined interventions	Moderate mechanistic and preclinical support for coordinated microbial, metabolic, and inflammatory effects	Plausible complementary strategy; interaction effects are not yet established
**ω-3 + probiotics**	Limited direct human evidence; outcomes are inconsistent and rarely designed to test interaction	Moderate mechanistic and preclinical support for lipid–microbe–immune interactions	Emerging combination with insufficient evidence for clinical claims
**Fiber + dates + probiotics**	No adequate direct human trials evaluating the full combination	Moderate mechanistic support based on a whole-food substrate plus selected microorganisms	Strong translational rationale for a date-based synbiotic, but currently hypothesis-generating
**Dates + fiber + ω-3/ω-6 + probiotics**	No adequate direct human trials evaluating all four components simultaneously	Network-level mechanistic support only	An integrative model for research, not an established therapeutic intervention

Direct human data indicate that a combination or closely corresponding intervention has been evaluated in humans with relevant outcomes; “mechanistic/preclinical” indicates that support is primarily derived from experimental models, indirect human findings, or pathway-level reasoning; “hypothetical” indicates that the proposed interaction has not been adequately tested in humans. The table highlights an important distinction between evidence for individual components and evidence for their interaction. Evidence that fiber supports fermentation, that probiotics can affect selected gastrointestinal outcomes, or that ω-3 fatty acids influence inflammatory signaling does not by itself prove that combining them produces interactive effects. Interaction claims require factorial or adequately powered comparative trials in which the individual and combined components are tested under controlled conditions [26,34,123].

## Data Availability

No new data were created or analyzed in this study. Data sharing is not applicable to this article.
